# Progress in Anæsthetics

**Published:** 1899-07-29

**Authors:** 


### Progress in Anæsthetics.

(*Continued from page* 281.)

Schleich's solutions for general anæsthesia have not met with any degree of success. After personal observations of 700 cases in which they were used, Dr. Rodman[7] makes, amongst others, the following unfavourable comments: During administration the reflexes are lost early, especially the conjunctival, thus depriving the anæsthetist of his most important safeguards; the pulse is slow, and cyanosis is present in the majority of cases; the pupils are somewhat dilated; if not carefully guarded against, general cyanosis, infrequent and shallow respirations, rapid pulse, and respiratory failure supervene, requiring energetic treatment. The after effects and discomfort are just as marked as after chloroform or ether, vomiting occurring to the same extent. The period of awakening is not shortened. Several of the patients developed bronchitis, followed in some cases by pneumonia. In three cases the urine, negative before operation, contained albumen and casts after it. Dr. Rodman thinks that the solutions are certainly less free from danger than ether or even chloroform.

Some very interesting experiments have been made by Doctors Thomas and Kemp[8] on the effects of different anæsthetics upon the circulation through the kidneys and the blood pressure generally. These experiments were carried out by placing the kidney of the animal experimented upon in an oncometer, whilst a glass cannula was inserted in the carotid. These instruments connected with the necessary accessory apparatus recorded the changes in the form of tracings upon smoked paper. The conclusions arrived at were, briefly, that ether produces a special contraction of the renal arterioles with a consequent damaging effect upon the renal secretory cells, similar to those which follow clamping the renal artery. The kidney shrinks in bulk, accompanied by diminution of secretion, marked albuminuria, and finally suppression. These facts contraindicate the use of ether when renal disease is present. The effect of chloroform upon the kidney is apparently nil, but its action on the heart, as shown by the carotid tracing, is directly depressing. The results with A. C. E. mixture varied very much with the method of administration. If administered with plenty of air the effect was similar to that of chloroform alone, but if administered as ether is when given alone, the combined effects of chloroform on the heart and ether upon the kidney manifested themselves, and if the anæsthetic was pushed such powerful effects upon the breathing and heart were produced that artificial respiration had to be resorted to. These observers do not see any advantage in the A. C. E. mixture, but rather the reverse. More injurious effects followed the administration of Schleich's anæsthetic even than those of the A. C. E. mixture.

In the matter of local anæsthetics the value of the infiltration anæsthesia of Schleich, the induction of an acute local œdema,[9] is receiving confirmation. Some serious major operations have been successfully performed by this method, including irreducible hernia, gastro-enterostomy, and others, the results being satisfactory. For smaller operations by local anæsthetics, Sudeck recommends Oberst's method of regional anæsthesia, which consists of paralysing the nerve trunks and all the anastomoses of a region by the injection of a 1 per cent. cocaine solution after previously connecting the parts. This is more especially applicable to operations on the fingers and toes, or heads of the metacarpal bones; though Mancz says it may be used in excision of the hand and foot. "No evil results have followed the application of cocaine in this way."

Eucaine B is more advantageous for ophthalmic work than cocaine,[9] because of its "inferior toxicity, its inactivity as regards the pupil and accommodation, its antiseptic action, and the greater stability of its solutions." Legrand recommends a 2 per cent. of eucaine B in general surgery. Wohlgemuth states the toxicity of eucaine to be greater than has been hitherto believed. Legrand has performed a number of operations, including a radical cure of hernia, under eucaine B. The following conclusions are given: "(1) Eucaine B is a good local anæsthetic; it is about three and a half times less toxic than cocaine, and it produces anæsthesia as rapidly and completely, but is of shorter duration. (2) In inflamed tissues it gives results as uncertain as cocaine. (3) It possesses a vaso-dilating action, which may give rise to certain inconveniences not obtained with cocaine. (4) It produces vomiting when applied to mucous surfaces or wounds, which usually lasts for 25 minutes, but may be maintained for one and a half hours." Legrand agrees with Schmitt that neither eucaine A or eucaine B is likely to replace cocaine.

Herzog[10] has given the results of some experiments undertaken on animals, with a view of testing the efficiency of Laborde's method of "rhythmical traction of the tongue" in cases of apparent death from anæsthetics. He administered chloroform to dogs until respiration had ceased for one and a half minutes. He found that Larborde's method was useless in cases of asphyxia in a late stage of narcosis, but in an early stage of narcosis Laborde's method was distinctly useful when associated with other forms of resuscitation. The rationale of traction upon the tongue is that it stimulates the centres in the medulla, which necessitates an increased supply to the part. The respiratory centre in close proximity also shares in the beneficial effect.

[7] Med. Rec., Oct. 1, 1898. [8] Med. Rec., Sep. 3, 1898. [9] Practitioner, Sept., 1899. [10] Brit. Med. Jour., July 16, 1899. [11] Clin. Rec., Jan. 11, 1899.

